# Mock community experiments can inform on the reliability of eDNA metabarcoding data: a case study on marine phytoplankton

**DOI:** 10.1038/s41598-023-47462-5

**Published:** 2023-11-17

**Authors:** Nadia Marinchel, Alexis Marchesini, Davide Nardi, Matteo Girardi, Silvia Casabianca, Cristiano Vernesi, Antonella Penna

**Affiliations:** 1 Department of Pure and Applied Sciences, University of Urbino, Urbino, Italy; 2 Department of Biomolecular Sciences, University of Urbino, Urbino, Italy; 3 Research Institute on Terrestrial Ecosystems (IRET), National Research Council (CNR), Porano, Italy; 4 National Biodiversity Future Center, Palermo, Italy; 5 DAFNAE, University of Padova, Legnaro, PD Italy; 6 Conservation Genomics Research Unit, Research and Innovation Centre, Fondazione Edmund Mach, S. Michele all’Adige, Italy; 7 Fano Marine Center, Inter-Institute Center for Research on Marine Biodiversity, Resources and Biotechnologies, Fano, Italy; 8 CoNISMa, National Inter-University Consortium for Marine Sciences, Rome, Italy; 9 Forest Ecology Unit, Research and Innovation Centre, Fondazione Edmund Mach, S. Michele all’Adige, Italy

**Keywords:** Ecology, Community ecology, Molecular ecology, Marine biology

## Abstract

Environmental DNA metabarcoding is increasingly implemented in biodiversity monitoring, including phytoplankton studies. Using 21 mock communities composed of seven unicellular diatom and dinoflagellate algae, assembled with different composition and abundance by controlling the number of cells, we tested the accuracy of an eDNA metabarcoding protocol in reconstructing patterns of alpha and beta diversity. This approach allowed us to directly evaluate both qualitative and quantitative metabarcoding estimates. Our results showed non-negligible rates (17–25%) of false negatives (i.e., failure to detect a taxon in a community where it was included), for three taxa. This led to a statistically significant underestimation of metabarcoding-derived alpha diversity (Wilcoxon *p* = 0.02), with the detected species richness being lower than expected (based on cell numbers) in 8/21 mock communities. Considering beta diversity, the correlation between metabarcoding-derived and expected community dissimilarities was significant but not strong (R^2^ = 0.41), indicating suboptimal accuracy of metabarcoding results. Average biovolume and rDNA gene copy number were estimated for the seven taxa, highlighting a potential, though not exhaustive, role of the latter in explaining the recorded biases. Our findings highlight the importance of mock communities for assessing the reliability of phytoplankton eDNA metabarcoding studies and identifying their limitations.

## Introduction

Phytoplankton is a diverse, polyphyletic group of mostly unicellular, photoautotrophic organisms, which plays a pivotal role for the marine ecosystems and the whole Earth’s biosphere^1, 2^. In marine environments, the prokaryotic group of cyanobacteria is often the most abundant numerically, but eukaryotic phytoplankton are the most significant in terms of ecosystem processes^3^. Among eukaryotic phytoplankton, diatoms and dinoflagellates are ones of the most abundant, diverse and ecologically significant groups; e.g., the peculiarity of diatoms is their siliceous skeleton, while most of dinoflagellates are characterized by the presence of two locomotory flagella. Diatoms and dinoflagellates are responsible for seasonal blooms in temperate and polar waters and play a key role in primary production^4^. Other important, often abundant microalgae groups are haptophytes and the small prasinophytes^5^. As a whole, phytoplankton is one of the world’s major oxygen producers and is also responsible for nearly 50% of global primary production^1, 2^. In addition of being the foundation of the aquatic food web, phytoplankton play a key role in biogeochemical cycles, being involved in the biological pump, that takes inorganic carbon (e.g., carbon dioxide) from the atmosphere, fixes it into organic matter through photosynthesis and finally deposits it in marine sediments^1, 2^. Due to this process of carbon fixation, marine phytoplankton also has a great impact on Earth’s climate^6^. For all these reasons, together with its fast and sensitive response to changes in the aquatic environment, phytoplankton is considered to be a good indicator of marine ecosystems^5, 7^. Thus, monitoring the structure and composition of phytoplankton communities may be crucial for assessing water quality, evaluating shifts in the trophic state and studying the response of marine ecosystems to ongoing climate and environmental changes^8, 9^. For example, in the Baltic Sea phytoplankton monitoring has been used to study the seasonal fluctuations in primary producers and their long-term role in nutrient and biogeochemical cycles, allowing the detection of environmental changes on a short to long timescale (from days to decades)^10^. Similarly, phytoplankton communities have been recently used to show the impact of raising temperature in the Mediterranean Sea^2, 11, 12^. Lastly, some phytoplankton species can produce dangerous biotoxins and in some situations can give rise to harmful algal blooms (HABs), that can cause problems for human health, alterations to marine ecosystems and economic losses to aquaculture and tourism activities^13^. Therefore, developing and implementing accurate, standardized and cost-effective methodologies for monitoring spatio-temporal trends of marine phytoplankton communities is a critical and urgent issue in many contexts^14^.

In the last decade, the new field of environmental DNA (eDNA) metabarcoding^15^, defined as the combination of DNA metabarcoding, i.e., the simultaneous identification of multiple taxa based on high-throughput sequencing (HTS) of a target DNA region, and environmental DNA, i.e., the direct extraction of genetic material from environmental samples, such as air, soil, sediment, water, etc., has emerged as a revolutionary approach to biodiversity research^16^. Indeed, eDNA metabarcoding represents a faster and relatively cheap alternative to conventional biodiversity assessment based on traditional sampling and morphological identification and has proven to be particularly effective in the detection of cryptic, rare and/or elusive species^17^. The metabarcoding of eukaryotic plankton communities by targeting 18S SSU rDNA regions is frequently used to assess diversity patterns from environmental samples^18–20^. In particular, the ribosomal V4 and V9 regions are popular choices for analysing communities of marine eukaryotic protists^21, 22^, due to their highly conserved primer binding regions and the ability to detect and identify a wide spectrum of phylogenetically distant taxa^17^. However, the 18S rDNA subunit’s gene copy number variation among various phytoplankton groups or even between individuals is a widely discussed issue, and many authors agree that it can be a cause of the discrepancy between true species abundance and metabarcoding-derived read counts introducing a bias in the latter^23–25^. In addition to the problems associated with the variability in rDNA gene copy numbers, a more general challenge in studies using amplicon-based metabarcoding is the choice of barcode region and primer pairs, which can limit or bias the estimated diversity, particularly when the target group of organisms is highly phylogenetically diverse^26^. Finding and applying appropriate markers is still a matter of debate for many metabarcoding applications, and primer bias, i.e. the preferential amplification of certain taxa over others, has become a well-known issue for several taxonomic groups. Indeed, sequence mismatches (even of a few base pairs) between primers and their target sequences, may result in the failure to detect some taxa or, together with PCR-induced biases, in skewed relative sequence abundances^17, 27, 28^. In addition, sub-optimal field (e.g., sample collection protocols), laboratory (e.g., DNA extraction) and bioinformatic procedures, including the use of incomplete or inaccurate reference sequence databases, and sequencing errors may be additional sources of error in metabarcoding studies^17^. All the above-mentioned shortcomings and problems of eDNA metabarcoding protocols can lead to false negatives, i.e. the failure to detect a taxon at sites (or in samples) where it is present, false positives, i.e. the detection of a taxon in a sample where it should actually be absent (i.e., not intentionally included)^29, 30^, or inaccuracies in quantitative estimates, due to the non-correspondence of read counts with the original abundances of taxa in the community/sample^31^. Therefore, eDNA metabarcoding results should be interpreted with caution and several authors are beginning to claim the need for context-specific validation studies^32–34^.

Mock communities, i.e., simulated communities of known and pre-defined species composition, provide an opportunity to test several of the above-mentioned uncertainties and can be used to assess the reliability of metabarcoding-derived results, both in terms of qualitative and quantitative data^35, 36^. The use of mock communities in preliminary studies can facilitate the development of more efficient and standardized protocols (from DNA extraction to primer selection and bioinformatic analysis), and their use as positive controls allows for the validation of eDNA studies, or at least can highlight their limitations.

To analyse and compare ecological communities, it is crucial to examine both the diversity of species within and among communities (alpha and beta diversity, respectively). The analysis of alpha and beta diversity levels allows to measure the spatio-temporal changes in community structure and composition, providing the information for the study of their biotic and abiotic drivers and for elucidating ecological processes operating at different scales, including biodiversity response to environmental stresses^10^. With the advent of eDNA metabarcoding and its application in biodiversity assessment, many researchers started to use the derived data as qualitative (number of retrieved taxa) and quantitative (number of amplicon read sequences) information for the calculation of alpha and beta diversity indices^18, 21, 37^, despite the uncertainties related to this methodological approach, as explained above. However, qualitative and quantitative biases potentially associated with metabarcoding data can lead to the non-detection of target species in biomonitoring or prevent the accurate reconstruction of the relationships between community composition and biotic and abiotic factors^29^.

In this study, we used mock communities to test the efficiency and accuracy of an eDNA metabarcoding protocol in reconstructing both alpha and beta phytoplankton diversity. We chose to adopt an amplicon sequencing approach, targeting the V4 region of the 18S SSU rDNA by means of a widely used broad-spectrum primer pair developed for microbial eukaryotic communities^38^. A total of 21 lab-generated mock communities, varying in species composition and abundance, were constructed using seven common and easily lab-cultured marine diatoms (*Chaetoceros socialis*, *Skeletonema marinoi*, *Thalassionema frauenfeldii* and *Pseudo-nitzschia* spp.) and dinoflagellates (*Alexandrium minutum*, *A. pacificum* and *Scrippsiella* spp.). As unicellular organisms, diatoms and dinoflagellates allowed us to assemble artificial communities directly based on the number of individuals (i.e., cells), instead of using DNA concentration, such as in the majority of mock community studies^39^, or more complex proxies for species richness^35^, thus allowing us to directly investigate the quantitative component of alpha and beta diversity. Using the 21 assembled mock communities and comparing metabarcoding-based and expected (computed from the number of cells included for the different taxa) composition and abundance estimates, this study aimed at: (i) evaluate the accuracy of the tested metabarcoding protocol in detecting the selected diatom and dinoflagellate taxa; (ii) assess the potential occurrence of false negatives (i.e., the failure to detect a taxon in a mock community in which it was included); and (iii) test the reliability of metabarcoding-derived alpha and beta diversity estimates computed from both qualitative and quantitative data. Based on the results obtained, we tried to investigate the possible explanations for the patterns that emerged.

## Results

### Primer testing and Sanger sequencing of the seven selected phytoplankton taxa

Sanger sequencing performed on DNA extracted from cultured cells before the assembly of mock communities, targeting the 18S V4 region using the same primer pairs as for metabarcoding, successfully amplified all the selected phytoplankton taxa and confirmed the taxonomic assignment of the cultured algal cells, ruling out potential misclassification in the preparatory steps of the experiment. The obtained sequences were deposited in GenBank (see Data Availability).

In silico PCR tests performed using FastPCR^40^ showed comparable, relatively high (96–97%), efficiency levels for the selected 18S V4 primers, in all the seven phytoplankton taxa included in our experiment, suggesting that preferential amplification due to primer bias should not be a major issue for the considered taxa.

Sanger sequencing performed using taxon-specific primer pairs, that targeted other ribosomal regions (5.8S and ITS), undoubtedly confirmed the presence of the DNA template of the taxa which resulted undetected by metabarcoding, in all mock communities where false negatives arose (see below section).

### Biovolume and 18S rDNA copy number determination

The estimated average cell biovolume, a morphometric descriptor of phytoplankton species, varied largely among the seven taxa included in the mock communities (42–26,434 µm^3^; Table 1), with average biovolumes for dinoflagellates largely exceeding those for diatoms (12,224 µm^3^ vs 151 µm^3^, respectively). The maximum average biovolume was recorded for *A. pacificum* (26,434 ± 1,675.43 µm^3^), followed by *A. minutum* (7,070 ± 404.58 µm^3^) and *Scrippsiella* spp. (3,167 ± 233.58 µm^3^). Among diatoms, *C. socialis* had the highest value (463 µm^3^), while *Pseudo-nitzschia* spp. (42 µm^3^ ± 4.21) had the lowest.

**Table 1:** Estimated average biovolumes and 18S rDNA copy number per cell for the different phytoplankton taxa included in the mock communities (SE = standard error; n.a. = data not available).

Taxonomic group	Taxon	Biovolume ± SE (µm^3^)	Mean 18S rDNA copy number/cell ± SE
Dinoflagellates	*Alexandrium minutum*	7,070 ± 404.58	10.11 ± 2.04
	*Alexandrium pacificum*	26,434 ± 1,675.43	1.1 ± 0.0005
	*Scrippsiella* spp.	3,167 ± 233.58	1.75 ± 0.13
Diatoms	*Chaetoceros socialis*	463 ± 95.91	1 ± 0.0002
	*Pseudo-nitzschia* spp.	42 ± 4.21	1 ± 0.0003
	*Skeletonema marinoi*	124 ± 14	4.06 ± 0.33
	*Thalassionema frauenfeldii*	288 ± 65.66	n.a

Similarly, the estimated values for the average 18S rDNA copy number per cell were variable (Table 1) ranging from one to about 10. Among dinoflagellates, *A. minutum* showed the highest 18S rDNA copy number (10 ± 2.04) followed by *Scrippsiella* spp. (1.75 ± 0.13) and *A. pacificum* (1.1 ± 0.0005), while among diatoms the only taxon with average copy number per cell > 1 was *S. marinoi* (4 ± 0.33).

### Mock community metabarcoding, species detection and false negatives

**Figure 1:**
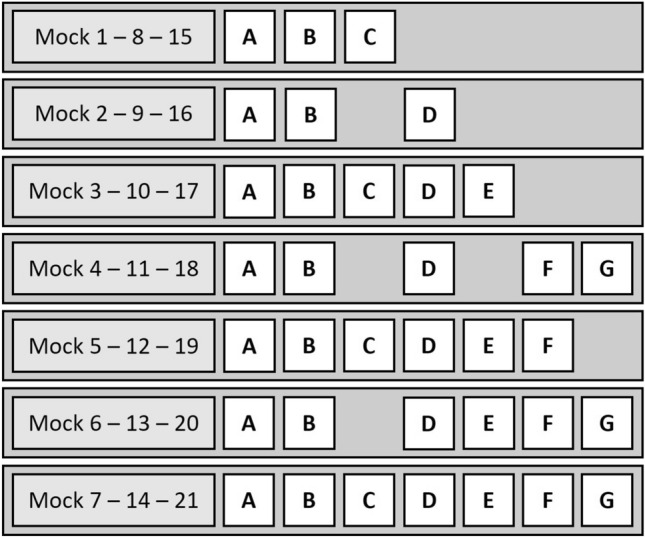
Schematic representation of the 21 phytoplankton mock communities. Each of the seven groups (see Materials and methods) is represented by three communities, which differ in taxa abundances (i.e., number of cells; see Table 2); A = *Alexandrium minutum*, B = *Chaetoceros socialis*; C = *Pseudo-nitzschia* spp., D = *Scrippsiella* spp., E = *Alexandrium pacificum*, F = *Skeletonema marinoi*, G = *Thalassionema frauenfeldii*.

The 18S rDNA amplicon sequencing of the 21 mock communities, assembled starting from lab-cultured cells of the seven diatom and dinoflagellate taxa and following the specific experimental design described in Table 2 and Fig. 1, yielded a total of 837,095 merged reads after trimming, corresponding to 111 ASVs (amplicon sequence variants, i.e. unique sequences). Of these, 830,930 reads (99.3%), corresponding to 50 ASVs, were clustered into seven OTUs, which resulted to be successfully classified into the seven target microalgal taxa. For each of the seven phytoplankton taxon included in the mock communities, the corresponding sequence IDs in the PR2 database (ver. 4.14.0) are reported in Table S1. The obtained classification was further checked by blasting the retrieved OTUs sequences in the NCBI Genbank database, to rule out the risk of misclassification due to potential bias in the reference database. The remaining 6,165 reads (0.7%), corresponding to 61 ASVs, remained unclassified and were manually assigned to non-target taxa (e.g., Insecta) with low sequence similarity or unclassified phytoplankton taxa (sequence similarity < 95%), after blasting their sequences with the NCBI Genbank database. All these sequences, most likely derived from minor contaminants or PCR artifacts, were removed from the dataset.

**Table 2:** Mock community experimental design.

Group	Mock	Dinoflagellates			Diatoms			
		*Alexandrium minutum* cell number	*Alexandrium pacificum* cell number	*Scrippsiella* spp. cell number	*Chaetoceros socialis* cell number	*Pseudo****-****nitzschia* spp. cell number	*Skeletonema marinoi* cell number	*Thalassionema frauenfeldii* cell number
1	1	3.3 × 10^5^	0	0	3.3 × 10^5^	3.3 × 10^5^	0	0
	2	3.3 × 10^5^	0	3.3 × 10^5^	3.3 × 10^5^	0	0	0
	3	2.0 × 10^5^	2.0 × 10^5^	2.0 × 10^5^	2.0 × 10^5^	2.0 × 10^5^	0	0
	4	2.0 × 10^5^	0	2.0 × 10^5^	2.0 × 10^5^	0	2.0 × 10^5^	2.0 × 10^5^
	5	1.6 × 10^5^	1.6 × 10^5^	1.6 × 10^5^	1.6 × 10^5^	1.6 × 10^5^	1.6 × 10^5^	0
	6	1.6 × 10^5^	1.6 × 10^5^	1.6 × 10^5^	1.6 × 10^5^	0	1.6 × 10^5^	1.6 × 10^5^
	7	1.4 × 10^5^	1.4 × 10^5^	1.4 × 10^5^	1.4 × 10^5^	1.4 × 10^5^	1.4 × 10^5^	1.4 × 10^5^
2	8	2.0 × 10^5^	0	0	4.0 × 10^5^	4.0 × 10^5^	0	0
	9	2.0 × 10^5^	0	4.0 × 10^5^	4.0 × 10^5^	0	0	0
	10	1.0 × 10^5^	1.0 × 10^5^	1.0 × 10^5^	3.5 × 10^5^	3.5 × 10^5^	0	0
	11	1.0 × 10^5^	0	1.0 × 10^5^	3.5 × 10^5^	0	3.5 × 10^5^	1.0 × 10^5^
	12	1.0 × 10^5^	1.0 × 10^5^	1.0 × 10^5^	3.0 × 10^5^	3.0 × 10^5^	1.0 × 10^5^	0
	13	1.0 × 10^5^	1.0 × 10^5^	1.0 × 10^5^	3.0 × 10^5^	0	3.0 × 10^5^	1.0 × 10^5^
	14	8.0 × 10^4^	8.0 × 10^4^	8.0 × 10^4^	3.0 × 10^5^	3.0 × 10^5^	8.0 × 10^4^	8.0 × 10^4^
3	15	4.0 × 10^5^	0	0	4.0 × 10^5^	2.0 × 10^5^	0	0
	16	4.0 × 10^5^	0	2.0 × 10^5^	4.0 × 10^5^	0	0	0
	17	3.5 × 10^5^	1.0 × 10^5^	1.0 × 10^5^	3.5 × 10^5^	1.0 × 10^5^	0	0
	18	3.5 × 10^5^	0	1.0 × 10^5^	3.5 × 10^5^	0	1.0 × 10^5^	1.0 × 10^5^
	19	3.0 × 10^5^	1.0 × 10^5^	1.0 × 10^5^	3.0 × 10^5^	1.0 × 10^5^	1.0 × 10^5^	0
	20	3.0 × 10^5^	1.0 × 10^5^	1.0 × 10^5^	3.0 × 10^5^	0	1.0 × 10^5^	1.0 × 10^5^
	21	3.0 × 10^5^	8.0 × 10^4^	8.0 × 10^4^	3.0 × 10^5^	8.0 × 10^4^	8.0 × 10^4^	8.0 × 10^4^

Taxon abundance (number of cells) was reported for each taxon and mock community. Mock communities were grouped according to the scheme described in Materials and methods.

**Figure 2:**
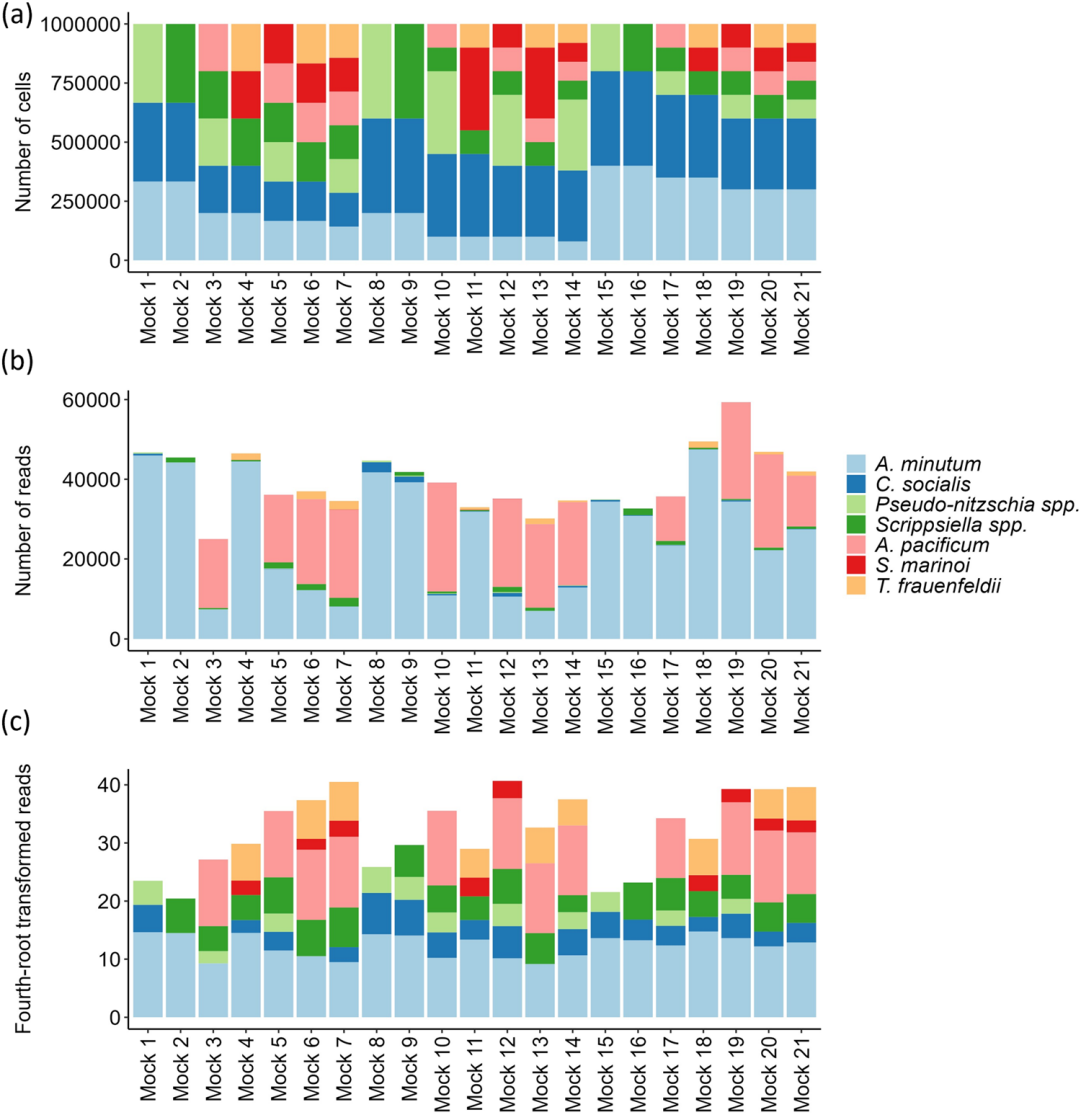
Stacked bar plots showing the composition of the 21 phytoplankton mock communities analysed; (**a**) expected composition (based on cell counts): for each community, the number of cells of each taxon included is shown, according to the colours reported in the legend; (**b**) metabarcoding-based estimated composition: for each community, the number of sequence reads recorded for each taxon is shown; (**c**) metabarcoding-based estimated composition, after fourth-root transformation of the read counts (see Materials and methods for details).

The total number of reads varied among mock communities, ranging from 25,009 to 59,335, although they were constructed starting from the same, predefined, total number of cells (1.0 × 10^6^). Considering the single algal taxa separately, *Alexandrium minutum* showed the highest mean number of reads in the 21 mock communities (2.64 × 10^4^), while *Skeletonema marinoi* showed the lowest average reads number of 1.98 × 10^1^ (Table 3). The sequenced negative control (extraction blank) resulted to be clean (0 reads detected for all the considered microalgal taxa). In general, the metabarcoding-derived community composition better reflected the expected composition (based on cell counts for each taxon) after applying the fourth-root transformation to read counts, as suggested to account for the intrinsic unevenness of metabarcoding data (see Materials and methods; subsection Bioinformatics and statistical analysis). In the communities obtained from the untransformed metabarcoding read counts (Fig. 2b), *A. minutum* and *A. pacificum* were overrepresented compared to their expected abundances (Fig. 2a), while all the other taxa resulted to be clearly underestimated. The above-described distortion of the estimated taxa abundances appeared to be mitigated after fourth-root transformation of read counts (Fig. 2c). Consequently, we decided to rely on the fourth-root transformed read counts data for further analysis.

**Table 3:** Species detection levels in the analysed mock communities.

Taxon	N	Mean cell number	Mean read number	Pearson R^a^	Ratio (R/C)	False negatives	False negatives > 100 reads
*Alexandrium minutum*	21	2.30 × 10^5^	2.64 × 10^4^	**0.63**	0.123	0	0
*Alexandrium pacificum*	12	6.84 × 10^4^	1.14 × 10^4^	**0.89**	0.181	0	0
*Scrippsiella* spp.	18	1.32 × 10^5^	7.39 × 10^2^	**0.62**	0.006	0	1
*Chaetoceros socialis*	21	3.02 × 10^5^	3.68 × 10^2^	**0.54**	0.001	4	8
*Pseudo-nitzschia* spp.	12	1.27 × 10^5^	8.14 × 10^1^	**0.78**	0.0004	2	7
*Skeletonema marinoi*	12	8.98 × 10^4^	1.98 × 10^1^	**0.55**	0.0002	3	11
*Thalassionema frauenfeldii*	8	5.09 × 10^4^	5.44 × 10^2^	**0.94**	0.0106	0	0

For each taxon, the overall number of expected occurrences (N, based on the pre-defined community compositions) was reported together with the mean cell number, the mean number of metabarcoding sequence reads, the Pearson’s correlation coefficients between number of cells and reads, the R/C ratio (total number of reads divided by total number of cells; see Materials and methods), and the number of false negatives (with and without the 100 reads cut-off).

^a^Statistically significant correlations were reported in bold.

Four out of seven microalgal taxa were always amplified in all the mock communities where cells of the considered taxa were included: *Alexandrium minutum*, *Alexandrium pacificum*, *Scrippsiella* spp. and *Thalassionema frauenfeldii*, while three taxa, i.e., *Chaetoceros socialis*, *Pseudo-nitzschia* spp. and *Skeletonema marinoi* occasionally gave rise to false negatives (i.e., the failure to detect a taxon in a mock community where it was expected based on the included algal cells).

Specifically, false negatives were detected in mock 2, 3, 6 and 13 for *C. socialis* (4/21 communities where it was included; 19%); in mock 5, 13 and 14 for *S. marinoi* (3/12 communities where it was included; 25%); and in mock 7 and 21 for *Pseudo-nitzschia* spp. (2/12 communities where it was included; 17%; see Table 3). Moreover, one false positive case was found for *Pseudo-nitzschia* spp., although with a very low number of reads (mock 9; 239 reads).

For all the taxa that occasionally gave rise to false negatives, the R/C ratio (defined as the ratio between total number of reads and total number of included cells; see Bioinformatics and statistical analysis subsection in Materials and methods), resulted to be ≤ 0.001.

When OTUs were filtered applying the 100 reads per sample cut-off threshold, to control for potential minor contaminations, sequencing errors, etc. (see Materials and methods for details), and therefore when adopting a less conservative definition for determining the presence of a taxon, also the dinoflagellate *Scrippsiella* spp. gave rise to a case of false negative, in addition to *C. socialis*, *Pseudo-nitzschia* spp. and *S. marinoi*. The R/C ratio for *Scrippsiella* spp. resulted to be 0.006, therefore close to the previously stated value of 0.001, characterizing the other taxa that occasionally reported false negatives. Moreover, the frequency of false negatives for *C. socialis*, *Pseudo-nitzschia* spp. and *S. marinoi* in the 21 communities increased by 100–300% (*C. socialis*: 8 false negatives; *S. marinoi*: 11; *Pseudo-nitzschia* spp.: 7). Therefore, adopting a less conservative definition for determining the presence of a taxon in each mock community (i.e., using the 100 reads cut-off) resulted in a higher number of false negatives.

Notably, the diatom *T. frauenfeldii* and the two dinoflagellates *A. pacificum* and *A. minutum,* which never gave rise to false negatives with or without the 100 reads cut-off threshold, showed a R/C ratio > 0.01 indicating an overall high sequencing yield.

For each diatom and dinoflagellate taxon, Pearson’s correlation test (Table 3) showed a significant and positive correlation between the number of cells included in the mock communities and the number of metabarcoding-derived reads (after fourth-root transformation; see Materials and methods). *T. frauenfeldii* and *A. pacificum* showed the strongest correlations (R^2^ = 0.94, R^2^ = 0.89, respectively; *p*-value < 0.001), whereas *S. marinoi* and *C. socialis* showed the weakest correlations (R^2^ = 0.55, R^2^ = 0.54, respectively; *p*-value < 0.001). Similar patterns were highlighted when repeating the correlation tests after normalization of read counts (i.e. total sum equal to one), with *T. frauenfeldii* and *A. pacificum* again showing the highest correlation coefficients and with the only difference consisting in the non-detection of a significant correlation for *Scrippsiella* spp. (although R^2^ = 0.41; Table S2).

### Alpha diversity

In the analysed mock communities, the estimated levels of species richness (i.e., computed from metabarcoding data after fourth-root transformation of read counts; see Materials and methods; average value: 4.62) were lower than the expected (i.e., computed based on the number of cells included for each taxon in the community; average value: 5) and this difference was statistically significant (average difference =  − 0.38; Wilcoxon signed rank test, z =  − 2.24, *p* = 0.02). Specifically, based on metabarcoding data, one of the included species was not detected in mock community 2, 3, 5, 6, 7, 14 and 21; while two of the included species were not detected in mock community 13 (Table 4), as a result of the false negatives cases discussed above. Only in one mock community, species richness was higher in the metabarcoding-based estimate than expected from the included cells, due to a false positive (*Pseudo-nitzschia* spp.; detected with a low number of read counts in mock 9, where no cells for the considered taxon were included). Similar patterns were recorded for Pielou’s evenness and Simpson’s index. An average, statistically significant difference of − 0.05 was observed between the estimated and the expected values for Pielou’s evenness (Wilcoxon signed rank test: z =  − 3.07, *p* < 0.01). The metabarcoding estimates indicated that the taxa were less evenly distributed than the expected (based on cell counts) in 14/21 mock communities (maximum recorded difference: − 0.16, in mock 1 and 4). An average difference of − 0.06 between estimated and expected values was recorded for Simpson’s index (Wilcoxon signed rank test: z =  − 3.29, *p* < 0.01). Based on metabarcoding data, 17/21 mock communities showed less diversity than expected (maximum observed difference: − 0.16, in mock 2).

**Table 4:** Alpha diversity indeces for each phytoplankton mock community.

	Expected richness	Estimated richness	Expected Pielou’s eveness	Estimated Pielou’s eveness	Expected Simpson's diversity index	Estimated Simpson's diversity index
Mock 1	3	3	1	0.84	0.67	0.54
Mock 2	3	2	1	0.87	0.67	0.41
Mock 3	5	4	1	0.88	0.80	0.67
Mock 4	5	5	1	0.84	0.80	0.69
Mock 5	6	5	1	0.91	0.83	0.75
Mock 6	6	5	1	0.92	0.83	0.75
Mock 7	7	6	1	0.93	0.86	0.79
Mock 8	3	3	0.96	0.90	0.64	0.59
Mock 9	3	4	0.96	0.91	0.64	0.68
Mock 10	5	5	0.89	0.92	0.73	0.74
Mock 11	5	5	0.89	0.89	0.73	0.71
Mock 12	6	6	0.92	0.94	0.78	0.79
Mock 13	6	4	0.92	0.96	0.78	0.72
Mock 14	7	6	0.89	0.91	0.79	0.78
Mock 15	3	3	0.96	0.83	0.64	0.53
Mock 16	3	3	0.96	0.88	0.64	0.57
Mock 17	5	5	0.89	0.90	0.73	0.74
Mock 18	5	5	0.89	0.86	0.73	0.69
Mock 19	6	6	0.92	0.87	0.78	0.75
Mock 20	6	6	0.92	0.89	0.78	0.76
Mock 21	7	6	0.89	0.91	0.79	0.78

Expected values correspond to those calculated based on the number of cells included for each taxon in the community; estimated values are those derived from the metabarcoding data, after fourth-root transformation of the read counts (see Bioinformatics and statistical analysis subsection, in Materials and methods).

### Beta diversity

**Figure 3:**
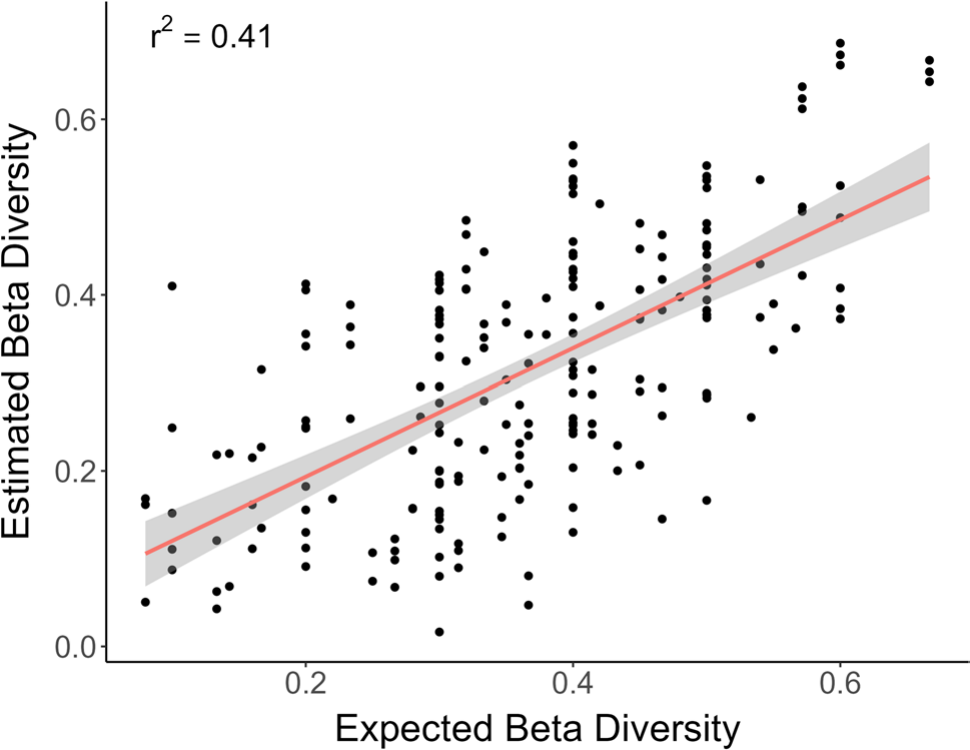
Linear regression of estimated beta diversity (based on metabarcoding read counts, after fourth root transformed read counts; see Materials and methods) vs expected beta diversity (based on cell counts).

Considering total beta diversity (βtot), multiple regression on distance matrices (MRM) highlighted a linear and moderate, positive relationship between the estimated (i.e., metabarcoding-derived) and expected (i.e., based on cell-counts) pairwise dissimilarity matrices (R^2^ = 0.41, *p* < 0.01) (Fig. 3). This result indicated that the dissimilarities between the 21 mock communities, assembled with different taxa composition and abundances (by means of controlling the number of cells per taxon), were captured by the metabarcoding-derived estimates, but the correspondence between the estimated and expected (cell-count based) beta diversity patterns was not perfect.

## Discussion

Marine phytoplankton includes a wide range of eukaryotic unicellular organisms, some of which can be grown in laboratory cultures, and are therefore representing an appropriate target group for the creation of mock communities with controlled species composition to test the efficacy of metabarcoding protocols.

In this study, we selected seven unicellular diatom and dinoflagellate taxa, which can be commonly found in marine coastal samples collected in the north-western Adriatic Sea, and constructed 21 mock communities of varying species composition and abundances (for more details on the selected taxa and the experimental design, see Materials and methods) by controlling the number of cells for each taxon according to a pre-defined experimental design. This allowed us to evaluate the species detection accuracy of an eDNA metabarcoding protocol, targeting the V4 region of 18S SSU rDNA (a common choice in eukaryotic phytoplankton metabarcoding^38^), and to directly compare estimated (i.e., derived from metabarcoding) with expected (i.e., computed from the cell numbers of the different taxa included in the communities) alpha and beta diversity patterns, both from qualitative (taxa composition) and quantitative (cell abundances) data.

Our main findings were: (a) low species detection rates for some of the seven phytoplankton taxa and, as a consequence, (b) biased metabarcoding estimates of alpha and beta diversity. Specifically, three of the seven microalgal taxa (*Chaetoceros socialis*, *Pseudo-nitzschia* spp. and *Skeletonema marinoi*) gave rise to non-negligible rates of false negatives in the metabarcoding results (i.e., no sequence reads were detected in mock communities where they were expected based on the algal cells included). The other four taxa, on the other hand, never gave rise to false negatives. Therefore, some taxa seemed to be more prone than others to generate false negatives with the metabarcoding protocol employed, and recovery of these taxa by means of Sanger sequencing, using taxon-specific primers, excluded laboratory errors in mock community preparation and DNA extraction as a possible explanation. Notably, one of the taxa exhibiting false negatives, i.e., *Pseudo-nitzschia* spp., actually includes also potentially toxic algae^41^.

Overall, false negatives in metabarcoding results were not strictly related to taxa abundances (i.e., number of cells) in the mock communities considered. Indeed, false negatives also occurred in mock communities where the considered taxon was included with high abundance of cells, such as in mock 13, where *C. socialis* and *S. marinoi* were both represented by 3 × 10^5^ cells, three times more abundant than all the other included taxa.

The discussed bias in species detection for some taxa, as well as biased abundance estimates, led to a significant, though not severe, underestimation of metabarcoding-derived alpha and beta diversity levels. The latter seemed to be more robust to bias in our case study, as estimated and expected (i.e., based on cell counts) community dissimilarities were moderately correlated; however, it should be noted that in more species-rich communities (such as in marine environmental samples) the effect of imperfect species detection could be more severe.

It should be noted that several precautions were taken to account for PCR stochasticity and the intrinsic unevenness of metabarcoding data: three independent PCR replicates were performed for each community and pooled together for sequencing, and fourth-root transformation was applied to read counts^19, 42^. The latter clearly resulted in a better correspondence between metabarcoding-derived and expected (based on cell counts) compositional data (see Fig. 2) but didn’t eliminate the discussed biases.

Primer bias, a frequent cause of false negatives in metabarcoding studies (see Introduction), seemed not to be the most likely explanation for the occasional detection failures in our experiment, since the chosen primer pair resulted in the same efficiency for all the seven phytoplankton sequences, after in silico PCR tests, and successfully amplified all the seven taxa when performing Sanger sequencing for taxonomic confirmation of the cultured cells. However, it should be noted that results of in silico and in vitro PCR can be somehow different, as the tuning of the PCR conditions can affect the performance of primers; moreover, amplification conditions can be different in single-taxon PCR compared to mock community metabarcoding PCR.

On the other hand, the calculated R/C ratio (i.e., for each taxon, the ratio between the total sum of read counts and the total sum of cells, in all communities) resulted to be related to the arising of false negatives: the taxa having the lowest R/C ratio values (*C. socialis*, *S. marinoi* and *Pseudo-nitzschia* spp.) corresponded with the taxa exhibiting the highest frequency of false negatives. This result may indicate that the overall frequency of false negatives for the different taxa in our experiments was related to the overall, taxon-specific, sequencing yield. Concordant evidence came from Pearson’s correlation test computed between the number of cells included in the mock communities and the number of metabarcoding-derived reads: although significant correlations were found for all taxa, they varied widely in magnitude and those with the strongest correlations, i.e., *Thalssionema frauenfeldii* and *Alexandrium pacificum* (R^2^ = 0.94 and R^2^ = 0.89, respectively; Table 3), never gave rise to false negatives, while those with the weakest correlations, i.e., *S. marinoi* and *C. socialis* (R^2^ = 0.55 and R^2^ = 0.54, respectively), corresponded to those with the highest frequency of false negatives in the metabarcoding results (three and four cases, respectively). In other words, when there was a strong correspondence between cell abundances and metabarcoding reads, no false negatives occurred; on the contrary, when the correspondence was weak (i.e., the same number of cells resulted in fewer sequence reads), false negatives occurred in some of the mock communities. These results were not surprising, but they clearly indicated that the arising of false negatives was not a stochastic process in our experiment.

In marine phytoplankton, several studies have raised the issue of rDNA gene copy number variation among different groups of organisms and species, highlighting it as a potential source of bias in metabarcoding results^18, 23, 25^, and several dinoflagellates are known to have large and repetitive genomes^43, 44^. Our qPCR-based estimates of the average 18S rDNA gene copy number per cell, highlighted *A. minutum* and *S. marinoi* as the taxa with highest value (approximately 10 and four, respectively), followed by *Scrippsiella* spp. and *A. pacificum*, with an average copy number per cell between one and two. The two dinoflagellates *A. minutum* and *A. pacificum* always dominated the communities in terms of read counts (Fig. 2c; Table S3 and Table S4); however, the mean number of read counts recorded for *S. marinoi* was relatively low. None of the four species with the highest average gene copy number gave rise to false negatives, except for *S. marinoi* (three false negatives out of 12 communities where it was included). On the other hand, the lowest values for 18S rDNA gene copy number were recorded for *C. socialis* and *Pseudo-nitzschia* spp. and both taxa gave rise to false negatives, in four and two communities, respectively. Therefore, our data indicated a relationship between low average 18S rDNA gene copy number, low detection rates and abundance bias for some taxa, but with the notable exception of *S. marinoi.* Moreover, it should be noted that the systematic overamplification of *A. pacificum*, clearly evident in the mean number of read counts, can be hardly attributed only to the 18S rDNA gene copy number, since the mean value assessed for this species was close to the taxa giving rise to false negatives. However, the nature of rDNA gene copy number variation can be multifaceted, complicating its estimation and introducing several sources of uncertainty. Indeed, numerous studies indicated both intra-specific and intra-individual variability in rDNA sequences, and both genetic and environmental factors have been reported as potential drivers influencing it^45^. Moreover, the cause of variation may be not only biological, but also due to experimental errors, such as PCR-induced mutations^46^.

Biovolume, a morphometric descriptor of phytoplankton cells, has been found to correlate with the relative abundance of rRNA genes in several, although not all, microalgae species^24, 47^. We therefore tried to investigate the effects of different biovolumes on the metabarcoding patterns recorded. Our estimates showed that *A. pacificum* and *A. minutum* had average biovolume values of 30 and 10 times bigger than other taxa, respectively (Table 1): these dinoflagellates had the highest yields in terms of overall sequence reads and they were always detected by the tested metabarcoding protocol when they were included in the community (i.e. no false negatives) (Table 3). On the other hand, the smallest biovolumes were measured for *Pseudo-nitzschia* spp. and *S. marinoi* and false negatives were recorded for both taxa (two and three cases, respectively). However, the taxon with the highest frequency of false negatives, i.e., the diatom *C. socialis,* showed an intermediate value for the average biovolume, higher than that of *T. frauenfeldii,* the diatom that never gave false negatives. Therefore, biovolume, which is sometimes used as a proxy for rDNA copy number, seemed to partially but not entirely explain the patterns observed.

Taken together, our results suggested a role for 18S rDNA gene copy number variation in biasing metabarcoding results, but not exhaustively and not for all taxa. In addition to the aforementioned uncertainties in 18S rDNA gene copy number estimation, which may act as a confounding factor, other potential explanations may be involved, such as imperfect DNA extraction for some taxa, the presence of species-specific PCR inhibitors and primer bias^48, 49^, although the latter was at least partially excluded in our experiment (see above). In some cases, another potential source of bias in 18S metabarcoding is the possible presence of secondary structures in the amplified region due to insertions/deletions or introns, with shorter and structurally simpler fragments being preferentially amplified over longer ones^29^. In order to disentangle the influence of gene copy number variation from all the other potential drivers, further experiments should be carried out, with specifically assembled mock communities that control for the above-mentioned factors.

It must be noted that with eDNA metabarcoding, several other factors not considered here, can give rise to false negatives and introduce errors into the results. In this experiment, we “sampled” whole, pre-defined communities and we were confident to sample all the taxa included (as a further check, this was confirmed by Sanger sequencing): therefore, sampling error was absent. However, for environmental samples, inappropriate sampling methods, environmental heterogeneity, low DNA persistence and other factors related to field sampling can play a major role in introducing uncertainties into the data. Moreover, suboptimal DNA extraction protocols and bioinformatics parameters, e.g. the adopted sequence identity threshold for OTUs clustering as well as inaccurate and incomplete reference sequence databases (or unequal representation of taxa) can be additional source of errors^30^. Again, due to the simplified nature of our experiment with low species richness communities and controlled conditions, we didn’t test different extraction protocols, nor different bioinformatic pipelines. Regarding the latter aspect, however, we manually inspected all the unclassified sequences (although being an almost negligible fraction of the total: 0.7%), focusing on the taxa that give rise to false negatives in our experiment. By comparing the unclassified sequences with the OTU sequences of the considered phytoplankton species, we excluded the possibility that their non detection was due to the clustering and classification parameters. It should be noted that some of the seven target taxa were underrepresented in term of corresponding sequences included in the PR2 reference database (see Table S1); however, the visual inspection of unclassified sequences ruled out also this possibility as potential explanation for the recorded false negatives and this was further confirmed by the fact that *Scrippsiella* spp. and *Thalassionema frauenfeldii*, the only two taxa represented by a single sequence in the PR2 database, did not gave rise to false negatives in our experiment (except for one case when applying the 100 reads cut-off threshold).

Nevertheless, in DNA metabarcoding from environmental samples, great attention should be paid to all the above-mentioned aspects related to sampling strategies and laboratory and bioinformatic protocols^30^.

Lastly, it is common practice in metabarcoding to apply post-processing filtering to ASV/OTU tables to account for the effects of false positives, potentially arising from weak cross-sample or exogenous contamination, PCR/sequencing errors and tag-jumps. As the sequenced negative control for our experiment was clean (0 reads for all of the target taxa), we decided to apply a fixed cut-off threshold and we chose the commonly used value of 100 reads (see subsection Bioinformatics and statistical analysis in Materials and methods, for a detailed explanation). The results from both unfiltered and filtered data were compared: when the 100 reads cut-off was applied to each community, false negatives increased in the three taxa already affected by this issue and an additional taxon gave rise to a false negative in one mock community. This is not surprising, given that (a) applying a cut-off based on the read counts implies a less conservative definition of presence/absence, and (b) the taxa affected by false negative issues correspond to those with the lowest yield in terms of number of reads (see above). Interestingly, we also found a false positive (i.e., detection of a taxon in a community where no cells for that taxon were included) in our metabarcoding results, although with a limited number of reads (possibly due to low cross-contamination of the sample). The potential occurrence of false positives is a well-known issues in eDNA metabarcoding and can affect results even when following strict sampling and laboratory procedures^50^; non-negligible rates of false positives had already been reported also for 18S metabarcoding of plankton mock communities^51^.

Given the simplified nature of our experiment, based on predefined communities with known composition, we were easily able to identify both false negative and false positive cases and our primary aim here was not to test the effect of different post-processing filtering strategies. However, in DNA metabarcoding from environmental samples, great attention should be paid to strategies for mitigating detection errors^29^. During the different steps of collection, extraction, amplification, and sequencing process, negative controls (i.e., blanks) should be included and sequenced to quantify the levels of contamination present; positive controls (i.e., samples which were already sequenced or with known composition) should be also included when possible. Moreover, rigorous post-processing error mitigation strategies should be carried out. Different approaches can be applied: in addition to the application of an arbitrary, fixed cut-off threshold based on read counts for ASVs/OTUs exclusion (which can vary according to the barcode region, target organisms and aims of the study; see e.g.^52, 53^, a proportional threshold based on relative frequencies (e.g., 0.01%, 0,1% or 1% ^52, 54^;) can be used, or more complex post-sequencing filtering pipelines, which rely on information from blanks to calculate and remove the contaminant reads for each ASV/OTU in each sample^55^. Lastly, when multiple replicates per sample/site are performed, site occupancy-detection models can be applied to assess the reliability of species detections in a probabilistic manner^42^.

## Conclusions

This study highlighted the weaknesses of a standard amplicon-based eDNA metabarcoding approach based on a single primer pair, when applied to a phylogenetically diverse group of organisms such as phytoplankton. The non-negligible rates of false negatives, recorded for some of the target taxa, warn us of the need to test the accuracy of species detection in phytoplankton eDNA metabarcoding protocols, prior to analyse environmental samples where species composition is unknown. Although this is obviously not possible for all the potentially present phytoplankton species, it is highly recommended particularly when one of the objectives is the monitoring of potentially toxic microalgae, which can cause harmful algal blooms^41^ with negative consequences for the marine environment, human health and the economy^56–59^. The frequency of these events is increasing due to climate change^60, 61^, and developing cost-effective and efficient strategies for monitoring potentially harmful algae is therefore becoming increasingly important. Notably, one of the taxa exhibiting false negatives in our experiment was *Pseudo-nitzschia* spp., which actually also includes potentially toxic species^41^.

Using a simplified experiment with controlled conditions, we showed how a metabarcoding protocol with suboptimal detection power, particularly when biased against some species, can lead to an underestimation of alpha and beta diversity. Biased estimates of alpha and beta diversity can limit our power to detect local extinctions, patterns of species turnover in space and time, and community nestedness^62^, making it difficult to accurately characterize spatio-temporal trends in phytoplankton communities and investigating their underlying ecological processes.

To partially mitigate some of the concerns raised, the use of multiple markers and/or primer sets^27, 36^ may be advisable in phytoplankton metabarcoding. Moreover, while Illumina MiSeq is still the most popular platform for amplicon sequencing, some studies showed that more modern platforms, such as the NovaSeq, may be able to detect more diversity (even at the same sequencing depth), therefore showing a higher detection power particularly for rare and/or low-abundance taxa^63^. Finally, it is worth mentioning that more recently developed PCR-free metabarcoding approaches, such as hybridisation capture techniques^64, 65^, could overcome the intrinsic limitations of amplicon-based metabarcoding approaches and potentially reduce the associated qualitative and quantitative biases.

As a general indication of good practice, we strongly recommend the use of mock community experiments in phytoplankton metabarcoding, particularly when large and expensive projects are planned or when monitoring potentially harmful algae. Mock communities should be used to set up and validate protocols, from DNA extraction to bioinformatics analysis, to control for potential biases and guide the interpretation of the resulting data, and can also be included as "positive controls" in routine analyses to assess the reproducibility of results. The assembly of mock communities based on cell counts or biomass (for multicellular organisms), rather than using DNA concentration, allows their use as standards to better control for abundance-related biases.

## Materials and methods

### Microalgal cultures, cell counts and biovolume determination

A total of seven marine phytoplankton taxa were used in this study, representing dinoflagellates and diatoms which can be commonly found in the Mediterranean Sea and can be easily grown in lab-cultures. Specifically, the selected taxa were the dinoflagellates *Alexandrium minutum* (strain CNR AMIV1), *Alexandrium pacificum* (strain CNR ACAT15P), *Scrippsiella* sp. (strain VGO1140) and the diatoms *Chaetoceros socialis* (strain CBA22), *Skeletonema marinoi* (strain CBA4), *Pseudo-nitzschia* spp. (strain CBAA2), *Thalassionema frauenfeldii* (strain CBA98), which were grown in F/2-Si and F/2 media^66^, respectively. Culture conditions were: 18 ± 1 °C and 20 ± 1 °C, for diatom and dinoflagellate taxa, respectively, under a standard 12:12 h light–dark cycle. The light was provided by cool-white, fluorescent bulbs (photon flux of 100 µE m^-2^ s^-1^). Cells were collected during the exponential growth phase and counted using an inverted microscope (Zeiss Axiovert 40 CFL, Germany) equipped with phase contrast according to the Utermöhl method^67^. Counting was carried out at × 200 or × 400 magnification on the entire Utermöhl chamber. Size measure of each taxon was done with an inverted microscope Nikon model Ti2-U (Tokyo, Japan). The average cell biovolume, a phytoplankton morphometric descriptor based on different cell features such as shape, length, diameter and height, was estimated for each of the seven diatom and dinoflagellate taxa by measuring morphometric parameters in 10 cells per taxon; calculations were made according to the principle of the most similar geometric shape^68^.

### Primer testing and Sanger sequencing of the seven selected phytoplankton taxa

For our metabarcoding experiment, we chose to amplify the V4 region of the 18S SSU rDNA using the TAReuk454FWD1 and TAReukREV3 primer pair (Table S5), specifically developed for broad-spectrum microbial eukaryotic communities^38^.

Before the assembly of mock communities, for each of the seven selected diatom and dinoflagellate taxa, cultured cells were collected and processed via Sanger sequencing, using the above mentioned primer pair, to confirm taxonomic assignment. For each taxon, genomic DNA was extracted from cultured cells using DNeasy Plant Kit (Qiagen, Hilden, Germany) following manufacturers’ instructions and the extracted DNA was stored at − 80 °C until molecular analyses. PCR amplification was carried out in a 50 µl reaction volume containing 1 × Reaction Buffer (Hot-Start Taq DNA Polymerase, 5U/µl, Biotechrabbit GmbH, Germany), 2 mM MgCl_2_, 1 × PCR Enhancer, 200 µm dNTP mix, 200 nm of each primer, 1U Taq DNA Polymerase and 10 µl of DNA volume. Thermocycling conditions were as follow: 95 °C for 2 min, followed by 12 cycles at 94 °C for 30 s; 57 °C for 45 s followed by 72 °C for 1 min; 28 cycles at 94 °C for 30 s, 45 °C for 45 s, 72 °C for 1 min, with a final extension at 72 °C for 1 min. PCR products were purified using the ExoSAP-IT kit (USB Corporation, Cleveland, OH). DNA sequencing reactions was performed following the ABI Prism Big-Dye Terminator Kit (Applied Biosystems) standard protocol and the sequencing reaction products were run on an ABI 3130xl Genetic Analyzer (Applied Biosystems). Raw sequences, after the removal of primer sequences, were edited and assembled in contigs using Sequencer 4.7 (Gene Codes Corporation, USA) and taxonomically assigned blasting their sequences with the NCBI Genbank database (National Center for Biotechnology Information; www.ncbi.nlm.nih.gov/genbank).

Prior to mock communities metabarcoding, in silico PCR were performed using FastPCR^40^, testing the selected 18S V4 primers on the sequences of the seven selected phytoplankton taxa (obtained as described above), with the aim of assessing primer efficiency for all the selected taxa.

For the taxa giving rise to false negatives in the metabarcoding results, the presence of template DNA in the mock communities was checked by Sanger sequencing using taxon-specific primers (Table S5): CsocF and CsocR for *C. socialis*, SkelssppF and SkelsppR for *S. marinoi*, Pseudo 5’ and Pseudo 3’ for *Pseudo-nitzschia* spp.^69^, with the following PCR thermal cycle conditions: initial denaturation 95 °C for 2 min; 35 cycles at 95 °C for 30 s, 28 °C for 30 s, 72 °C for 30 s; final extension at 72 °C for 5 min. For this specific aim, we decided not to use the same universal 18S primers chosen for mock community metabarcoding, but instead a set of already tested taxon-specific primers, because our primary aim here was to rule out completely the possibility that the detected false negatives could be due to potential errors in laboratory procedure during mock community assembly. Therefore, we wanted to avoid any potential risk of primer bias which, at least in theory, could derive from the use of less specific primers (though this possibility seems unlikely based on the results of our in silico test; see above).

### Quantification of average 18S rDNA copy number

In order to quantify the average 18S rDNA copy number per cell for each of the selected diatom and dinoflagellate taxon, real time PCR was performed, using class-specific primers targeting the 18S rDNA gene (Table S5): 1209f and Diatom18SR1 for diatoms; EUK528f and Dino18SR1 for dinoflagellates^47^. PCR reactions were carried out in a total volume of 25 µl, containing 1 × Hot Rescue Real Time master mix (Hot-Rescue Real Time PCR Kit-SG, Diatheva, Italy), 2 mM MgCl_2_, 300 nM each primer for both assays, 0.625 U of Hot-Rescue Plus DNA Polymerase, and 1 µl of undiluted and 1:10 diluted sub-samples of DNA. The qPCRs were carried out using the StepOne Real-Time PCR System (Applied Biosystems, Foster City, CA, USA) with different thermal cycle programs for the two phytoplankton assays^69^. The determination of rDNA copy number per cell was achieved by means of standard curve method described in Casabianca et al.^69^.

### Mock community experimental design

A total of 21 mock communities were prepared using the seven selected unicellular microalgae, following the experimental design described in Table 2 and Fig. 1.

The algal communities were assembled based on pre-defined different composition and taxa abundances (i.e., number of cells), for testing both qualitative (based on presence/absence) and quantitative (based on taxa abundances). The total number of cells per community was kept constant at a value of 1.0 × 10^6^ cells. This value roughly corresponds to the average phytoplankton cell concentration in surface seawater samples collected in the NW Adriatic Sea by means of Niskin bottles (personal observation; see also Neri et al.^2^, Totti et al.^70^) and it was chosen to simulate an example of environmental conditions (although they can vary largely). The 21 assembled mock communities can be ideally divided into three groups, each composed of seven communities (Table 2). In Group 1 (mock 1–7), the different taxa were included in the community with the same abundance (number of cells), but communities differed in species richness (i.e. number of taxa). In Group 2 (mock 8–14), two taxa were included in the communities with higher abundances than the others, one of these being the dinoflagellate *A. minutum*, a species with hypothesized amplification bias; communities also differed in species richness. In Group 3 (mock 15–21), communities were assembled following the scheme of Group 2, but *A. minutum* was included with lower abundance compared to all other taxa. The dinoflagellate *A. minutum* was identified as a candidate species to test for the effects of amplification bias, since this taxon showed the highest average 18S rDNA gene copy number based on qPCR quantification (see Table 1): for this reason, it was included in all mock communities with varying abundance combinations. *C. socialis*, one of the species exhibiting the lowest value for average 18S rDNA gene copy number, was also included in all mock communities with varying abundance combinations, with the purpose of testing how the two extremes (high and low 18S rDNA gene copy numbers) behave in different combinations.

Each mock community was prepared by mixing the required cell culture volumes in a 50 ml sterile falcon tube, and centrifuging them at 4000 rpm for 10 min. After removing supernatant, the pellets were stored at − 80 °C until DNA extraction.

### Mock community metabarcoding: DNA extraction, amplification and sequencing

Genomic DNA was extracted from the 21 assembled phytoplankton mock communities using DNeasy Plant Kit (Qiagen, Hilden, Germany) following manufacturers’ instructions and the extracted DNA was stored at − 80 °C until molecular analyses. One negative control (extraction blank) was included in the extraction batch, to test for DNA carry-over during the extraction process.

For amplicon sequencing, the V4 region of the 18S SSU rDNA was amplified using the TAReuk454FWD1 and TAReukREV3 primer pair (Table S5), and PCR amplification was performed under the same conditions as for Sanger sequencing (see previous section). A PCR negative control (PCR blank) was added to each PCR reaction batch, to check for potential contaminations. For each mock community, three independent PCR replicates were performed and then pooled together in a single sample for sequencing, to correct for the effect of PCR stochasticity. Each PCR product (including blanks) was checked on a 1.5% (w/v) agarose gel and visualized using a Gel Doc System (Bio-Rad). Extraction and PCR blanks showed no bands on gel, but we decided to proceed with the sequencing of the first, as a further check. Pooled PCR products were purified using the MinElute PCR Gel Extraction Kit (Qiagen, Hilden, Germany), following manufacturer’s protocol. Library preparation was performed by means of two amplification steps: an initial PCR amplification using specific primers and a subsequent amplification that integrates relevant flow-cell binding domains and unique indices (using the NexteraXT Index Kit; FC‐131‐1001/FC‐131‐1002). The obtained libraries were sequenced at Fondazione Edmund Mach (S. Michele a./A., Trento, Italy), using a MiSeq sequencing platform (Illumina, San Diego, CA) with a 2 × 300 bp paired-end mode.

### Bioinformatics and statistical analysis

Demultiplexed fastq sequences were checked for sequencing quality using FastQC v0.12^71^ and trimmed with Cutadapt version 3.5^72^. Primer sequences and their reverse complements were trimmed in both directions, from 5’ to 3' using the "-g" and "-G" parameters and from 3’ to 5’ using the "-A" and "-a" parameters, with the default maximum error rate of 0.1; sequences with a length shorter than 80 base pair were filtered out using the -m flag. Trimmed reads were then imported in QIIME2^73^, which was used for quality filtering (minimum per base quality threshold set to 30), denoising, merging and classification. Denoising was conducted using DADA2^74^ through the q2-dada2 plugin, with the following parameters: –p-trunc-len-f 295 and –p-trunc-len-r 220. The obtained Amplicon Sequence Variants (ASVs) were clustered into OTUs with a 97% similarity threshold and classified using the VSEARCH algorithm^75^ and the PR2 Protist Ribosomal Reference database, ver. 4.14.0^76^. OTUs with a percentage of identity < 97% were labelled as unassigned and, after a further check blasting their sequences with the NCBI Genbank database (National Center for Biotechnology Information; www.ncbi.nlm.nih.gov/genbank), removed from the dataset.

To account for the effects of false positives, potentially arising from weak cross-sample or exogenous contamination, PCR/sequencing errors and tag-jump, we applied a cut-off based on a fixed read counts threshold: when an OTU was present in a sample with number of reads < 100, it was removed from that sample. Although more complex filtering methods are available, considering the peculiar features of our study system (based on a low number of taxa and communities with pre-defined composition, which can be viewed as "intrinsic positive controls"), we decided to adopt the simplest strategy. The applied cut-off value of 100 reads per sample corresponds to a common choice in eDNA metabarcoding studies targeting different barcode regions (see e.g.,^52^, including 18S^77, 78^, to exclude false positives due to contaminations in reagents or cross-contamination of samples, or to exclude eDNA molecules that can still be present in the water environment but not deriving from living organisms present at the place and time of collection (see e.g., Kelly et al.^79^). It is worth mentioning that a less stringent threshold (e.g., 50 or 10 reads per sample–taxon; e.g., Esenkulova et al.^80^ can be used, but we opted for a relatively high one since this approach is considered more appropriate for reliable ecological inferences^54^. It should be noted that more accurate post-sequencing strategies (see Discussion) should be applied to mitigate detection errors in metabarcoding results from environmental samples, which are characterized, contrary to mock communities, by unknown species composition. Results from both filtered (cut-off: 100) and unfiltered data were then compared. For each taxon, we calculated the ratio between the obtained number of reads and the number of cells which were included in the mock communities (R/C ratio), as a raw estimate of the taxon-specific sequencing yield.

We computed both estimated (from metabarcoding data) and expected (based on cell counts of the different taxa) alpha diversity using the R package vegan, version 2.6–4^81^, by means of the following indices: species richness (function *specnumber*), Simpson's Diversity Index and Shannon’s Entropy Index (function *diversity*). In addition, Pielou’s evenness was calculated as H/log(S), where H is the Shannon’s entropy, and S is the total number of species (i.e. species richness). In order to compare the estimated (metabarcoding-based) and expected (cell count-based) alpha diversity levels, we used Wilcoxon signed rank test, to account for the non-normal distribution of data, using the R function *wilcox.test*. We then computed the total beta diversity (β_tot_) among mock communities, obtained by the sum of the species replacement and species richness components, using bat package, ver. 2.9.2^82^. Matrices of pairwise dissimilarity for total beta diversity among sites were computed using the function *beta*, for both estimated (metabarcoding-based) and expected (cell count-based) data. On the obtained pairwise dissimilarity matrices, multiple regression on distance matrix (MRM) was then applied in order to test the relationship between estimated and expected beta diversity, using the *MRM* function of R package ecodist, version 2.0.9^83^. Statistical analyses were conducted in R, ver. 4.1.0. 2021.05.18^84^ and Rstudio ver. 2022.12.03^85^; graphs were created using ggplot2 package ver. 3.4.0^86^.

For the graphical representation of compositional data and for the computation of correlation tests and alpha and beta diversity estimates, read counts were transformed using the fourth-root method with *tran* function of R package analogue, ver. 0.17–6^87^. This transformation was suggested when comparing metabarcoding read counts with other quantities, to account for the exponential nature of the PCR^19^.

### Supplementary Information

Supplementary Tables.

## Data Availability

The sequences obtained via Sanger sequencing for each of the seven selected phytoplankton taxon are deposited in NCBI GenBank under accession numbers OR752866-OR752872. The raw sequence data for the DNA metabarcoding experiment are deposited in the NCBI Sequence Read Archive (Bioproject accession PRJNA956448). An R markdown file with the R code used for the statistical analysis, together with the associated input files (OTUs table, cell counts, etc.) are available in the Mendeley Data repository at https://data.mendeley.com/datasets/hkh7nmdbrv/2 (doi: 10.17632/hkh7nmdbrv.2).
