# Supplementary material for: Mock community experiments can inform on the reliability of eDNA metabarcoding data: a case study on marine phytoplankton
Source: Sci Rep. 2023 Nov 17;13:20164. doi: 10.1038/s41598-023-47462-5 (PMC10656442; doi:10.1038/s41598-023-47462-5)
Supplement: Supplementary file 1 — Supplementary Tables. [file 41598_2023_47462_MOESM1_ESM.pdf]

Mock community experiments can inform on the reliability of eDNA metabarcoding data: a case study on marine phytoplankton

Nadia Marinchel[1,2*], Alexis Marchesini[3,4], Davide Nardi[5], Matteo Girardi[6], Silvia Casabianca[2,7,8], Cristiano Vernesi[4,9], Antonella Penna[2,7,8*]

[1]Department of Pure and Applied Sciences, University of Urbino, Urbino, Italy.

[2]Department of Biomolecular Sciences, University of Urbino, Urbino, Italy.

[3]Research Institute on Terrestrial Ecosystems (IRET), National Research Council (CNR), Porano, Italy

[4]National Biodiversity Future Center, Palermo, Italy

[5]DAFNAE, University of Padova, Legnaro (PD), Italy

[6]Conservation Genomics Research Unit, Research and Innovation Centre, Fondazione Edmund Mach, S. Michele all'Adige, Italy

[7]Fano Marine Center, Inter-Institute Center for Research on Marine Biodiversity, Resources and Biotechnologies, Fano, Italy

[8]CoNISMa, National Inter-University Consortium for Marine Sciences, Roma, Italy

[9]Forest Ecology Unit, Research and Innovation Centre, Fondazione Edmund Mach, S. Michele all'Adige, Italy

*Corresponding authors:

n.marinchel@campus.uniurb.it

antonella.penna@uniurb.it

Supplementary Information

Table S1. Sequence IDs in the reference sequence database. For each of the seven phytoplankton taxon included in the mock communities, the corresponding sequence ID in the PR2 database (ver. 4.14.0) is reported.

**Sequence ID in PR2**	**Taxon in mock communities**
32ebf7dcafc292eeb92689b17c509aa0	*Alexandrium minutum*
3eee971466aaeb80b51c3c8f071f8bd7	*Alexandrium minutum*
4c7782d100d3e2e86d970546b0a7a243	*Alexandrium minutum*
4d2132614927cfbbbb766e4b9f446298	*Alexandrium minutum*
55092fd7775beee0ff578642fdb20099	*Alexandrium minutum*
7499bc009de93376f99de94f2553d1f0	*Alexandrium minutum*
9305856d98cd257b4bb1ad217eabc342	*Alexandrium minutum*
0844526abcba8b62f42e2595201ebd19	*Alexandrium pacificum*
2f100273c6bf17e855c206addc06d1a7	*Alexandrium pacificum*
3e894c7fd6b3327cc8917042df221f13	*Alexandrium pacificum*
ad56748e9a82017f70310c3d15fdcd08	*Alexandrium pacificum*
c7c045c8671bffa301e77aae27bb8248	*Alexandrium pacificum*
da7275ae88ac4dfe73b75acd2b5c11f0	*Alexandrium pacificum*
ec3ce9be262fa5af4a2015876ed98685	*Scrippsiella* spp.
9d31c8fbece3dab87198a8698771fef1	*Thalassionema frauenfeldii*
47ed085d16d39c1ff1636dfe7d26ba7a	*Chaetoceros socialis*
5d04f614c12b856999693e89e749964b	*Chaetoceros socialis*
f65433e6bb89335b5a27b7a6a54e5e4f	*Chaetoceros socialis*
8180e9f10232a0eb0acc4f31d13bbb69	*Skeletonema marinoi*
cfeb0e3c701fa4dd64b83afffcb0cd36	*Skeletonema marinoi*
48c960e03cfb7f895b3c1a4ecc49178d	*Pseudo-nitzschia* spp.
2aabf93c6665a30e453b3b97b0102428	*Pseudo-nitzschia* spp.

Table S2. Pearson's correlation tests between number of cells and normalized read counts. For each taxon**,** the Pearson's correlation between the number of cells included in the mock communities and the number of DNA metabarcoding read counts after normalization (i.e., total sum equal to 1).

Taxon	Pearson R[a]
Alexandrium minutum	**0.60**
Alexandrium pacificum	**0.89**
Scrippsiella spp.	0.41
Chaetoceros socialis	**0.47**
Pseudo-nitzschia spp.	**0.60**
Skeletonema marinoi	**0.74**
Thalassionema frauenfeldii	**0.88**

[a] statistically significant correlations ($p < 0.05$) were reported in bold.

Table S3. Metabarcoding results. Number of read counts for each diatom and dinoflagellate taxon in the 21 mock communities. When a taxon was included in the mock community but resulted to be undetected by metabarcoding (i.e., false negative), this was reported as "0"; when no cells for a taxon were included in the community and no reads were found in metabarcoding results, this was reported as "-". A false positive case (i.e. recovering by metabarcoding of a taxon when this was not included in the community) is highlighted in bold. K represents the sequenced extraction negative control (blank; 0 reads were detected for all the considered phytoplankton taxa).

**Mock No.**	***Alexandrium minutum***	***Chaetoceros socialis***	***Pseudo-nitzschia* spp.**	***Scrippsiella* spp.**	***Alexandrium pacificum***	***Skeletonema marinoi***	***Thalassionema frauenfeldii***
1	45957	491	293	-	-	-	-
2	44218	0	-	1236	-	-	-
3	7414	0	20	341	17234	-	-
4	44475	24	-	344	-	38	1614
5	17433	108	99	1506	16974	0	-
6	12261	0	-	1530	21197	12	1975
7	8122	44	0	2164	22015	59	2147
8	41735	2541	404	-	-	-	-
9	39245	1431	**239**	916	-	-	-
10	10938	369	139	468	27266	-	-
11	31873	130	-	274	-	109	598
12	10623	942	216	1305	21933	79	-
13	7061	0	-	797	20880	0	1426
14	12918	413	73	73	20796	0	408
15	34389	419	136	-	-	-	-
16	30838	161	-	1658	-	-	-
17	23355	129	49	985	11171	-	-
18	47472	40	-	387	-	56	1537
19	34414	314	42	293	24245	27	-
20	22161	42	-	642	23369	18	656
21	27417	131	0	605	12720	18	1071
K	-	-	-	-	-	-	-

Table S4. Fourth-root transformed metabarcoding results. Number of read counts for each diatom and dinoflagellate taxon in the 21 mock communities, after fourth-root transformation (see Material and methods for details). When a taxon was included in the mock community but resulted to be undetected by metabarcoding (i.e., false negative), this was reported as “0”; when no cells for a taxon were included in the community and no reads were found in metabarcoding results, this was reported as “-“. A false positive case (i.e. recovering by metabarcoding of a taxon when this was not included in the community) is highlighted in bold. K represents the sequenced extraction negative control (blank; 0 reads were detected for all the considered phytoplankton taxa).

**Mock No.**	***Alexandrium minutum***	***Chaetoceros socialis***	***Pseudo-nitzschia* spp.**	***Scrippsiella* spp.**	***Alexandrium pacificum***	***Skeletonema marinoi***	***Thalassionema frauenfeldii***
1	14.64	4.71	4.14	-	-	-	-
2	14.5	0	-	5.93	-	-	-
3	9.28	0	2.11	4.3	11.46	-	-
4	14.52	2.21	0	4.31	0	2.48	6.34
5	11.49	3.22	3.15	6.23	11.41	0	-
6	10.52	0	-	6.25	12.07	1.86	6.67
7	9.49	2.58	0	6.82	12.18	2.77	6.81
8	14.29	7.1	4.48	-	-	-	-
9	14.07	6.15	**3.93**	5.5	-	-	-
10	10.23	4.38	3.43	4.65	12.85	-	-
11	13.36	3.38	-	4.07	-	3.23	4.95
12	10.15	5.54	3.83	6.01	12.17	2.98	-
13	9.17	0	-	5.31	12.02	0	6.15
14	10.66	4.51	2.92	2.92	12.01	0	4.49
15	13.62	4.52	3.41	-	-	-	-
16	13.25	3.56	-	6.38	-	-	-
17	12.36	3.37	2.65	5.6	10.28	-	-
18	14.76	2.51	-	4.44	-	2.74	6.26
19	13.62	4.21	2.55	4.14	12.48	2.28	-
20	12.2	2.55	-	5.03	12.36	2.06	5.06
21	12.87	3.38	0	4.96	10.62	2.06	5.72
K	-	-	-	-	-	-	-

Table S5. **Details of primer pairs used in this study.** Names and sequences of all the primer pairs used in this study, for: (a) Sanger sequencing for confirmation of taxonomic assignment of cultured cells prior to mock communities assembly, and eDNA metabarcoding of the assembled mock communities; (b) Sanger sequencing for taxa detection using species-specific primers (performed when a taxon included in the community resulted undetected from metabarcoding, i.e., false negative); and (c) quantification of the 18S rDNA gene copy number through real-time PCR.

Primer Name	Primer Sequence	Illumina adapters	Aim
TAReuk454FWD1[1]	5'-CCAGCASCYGCGGTAATTCC-3'	5'-GTCTCGTGGGCTCGGAGATGTGTATAAGAGACAG-3'	(a)
TAReukREV3[1]	5'-ACTTTCGTTCTTGATYRA-3'	5'-TCGTCGGCAGCGTCAGATGTGTATAAGAGACAGG-3'	(a)
CsocF[2]	5'-GGAGCGTCTGAGTATGGTCGT-3'	/	(b) for *C. socialis*
CsocR[2]	5'-GCCATCCAGACGCAAAAGTGT-3'	/	(b) for *C. socialis*
SkelsppF[2]	5'-CGATACACTGGTAGCGAGCC-3'	/	(b) for *Skeletonema* spp.
SkelsppR[2]	5'-TTCAGTTCGGTAATGGGCGG-3'	/	(b) for *Skeletonema* spp.
Pseudo 5'[2]	5'-CGATACGTAATGCGAATTGCAA-3'	/	(b) for *Pseudo-nitzschia* spp.
Pseudo 3'[2]	5'-GTGGGATCCRCAGACACTCAGA-3'	/	(b) for *Pseudo-nitzschia* spp.
1209f[3]	5'-CAGGTCTGTGATGC CCTT-3'	/	(c) for diatoms
Diatom18SR1[3]	5'-CAATGCAGWTTGATG AWCTG-3'	/	(c) for diatoms
EUK528f[3]	5'-CCGCGGTAATTCCA GCTC-3'	/	(c) for dinoflagellates
Dino18SR1[3]	5'-GAGCCAGATRCDCA CCCA-3'	/	(c) for dinoflagellates

[1] Stoeck, T., *et al*. Multiple marker parallel tag environmental DNA sequencing reveals a highly com-plex eukaryotic community in marine anoxic water. *Mol. Ecol.* **19**, 21–31 (2010).

[2] Casabianca, S., *et al*. Structure and environmental drivers of phytoplanktonic resting stage assemblages in the central Mediterranean Sea. *Mar. Ecol. Prog. Ser.* **639**, 73–89 (2020).

[3] Godhe, A., *et al*. Quantification of diatom and dinoflagellate biomasses in coastal marine seawater samples by real-time PCR. *Appl. Environ. Microbiol.* **74**, 7174–7182 (2008).
